# Commentary: Effect of probiotics at different intervention time on glycemic control in patients with type 2 diabetes mellitus: a systematic review and meta-analysis

**DOI:** 10.3389/fendo.2025.1514969

**Published:** 2025-03-19

**Authors:** Shanshan Wu, Yanhai Wang

**Affiliations:** ^1^ Clinical Laboratory Department, The Affiliated Hospital of Liaoning University of Traditional Chinese Medicine, Shenyang, Liaoning, China; ^2^ Department of Laboratory Medicine, Sichuan Provincial People’s Hospital Chuandong Hospital & Dazhou First People’s Hospital, Dazhou, Sichuan, China; ^3^ Clinical Laboratory Department, Hohhot First Hospital, Hohhot, Inner Mongolia Autonomous Region, China

**Keywords:** type 2 diabetes mellitus, diabetes mellitus, probiotics, fasting blood glucose, HbA1c, body mass index, insulin and HOMA-IR

I would like to present my views on the article titled “*Effect of Probiotics at Different Intervention Times on Glycemic Control in Patients with Type 2 Diabetes Mellitus: A Systematic Review and Meta-Analysis*,” authored by Wang et al. (1). During my review of the study, I identified several data entry errors that have affected the conclusions of the corresponding analyses. For example, the results of our re-analysis after data correction showed that patients with T2DM who took probiotics for 12-24 weeks had a more significant decrease in BMI compared to the placebo group. However, in the study by Wang et al. (1), the aforementioned results were not statistically significant. In the interest of scientific accuracy and to prevent further confusion, I propose the following corrections:

1. Supplementary Figure 3A (1): In the study by Asemi (2013) (2), the SD values should be 52.76 and 57.15 for the probiotic and placebo groups, respectively. Similarly, in the study by Mazloom (2013) (3), the SD values should be 60.9 and 65.13 for the probiotic and placebo groups, respectively. The aforementioned study reports SE, whereas SD needs to be calculated through SE, the detailed calculation methods are outlined in the statistical analysis section.
2. Supplementary Figure 3B (1): In Asemi’s study (2), the SD values should be 2.03 and 1.51 for the probiotic and placebo groups, respectively. For the study by Tonucci (2017) (4), the mean and SD values should be -0.67 and 1.46 in the probiotic group, and 0.31 and 1.17 in the placebo group. Detailed calculations are described in the statistical analysis section.
3. Supplementary Figure 3C (1): For Asemi (2013) (2), the SD values should be 5.15 and 6.91 for the probiotic and placebo groups, respectively. Similarly, in Mazloom’s study (3), the SD values should be 0.57 and 0.08 for the probiotic and placebo groups, respectively. For Tonucci (2017) (4), the mean and SD values should be -0.7 and 4.79 in the probiotic group, and -1.65 and 4.25 in the placebo group. Detailed calculations are provided in the statistical analysis section.
4. Supplementary Figure 3D (1): For Asemi (2013) (2), the SD values should be 2.06 and 3.96 for the probiotic and placebo groups, respectively. In Mazloom (2013) (3), the SD values should be 4.4 and 1.32 for the probiotic and placebo groups, respectively. For Tonucci (2017) (4), the mean and SD values should be 0.02 and 1.68 in the probiotic group, and 0.15 and 1.21 in the placebo group. Detailed calculations are provided in the statistical analysis section.
5. Corrections in Subgroup Analysis: In Supplementary Figure 4A (1), for Asemi (2013) (2), the SD values should be 52.76 and 57.15 in the probiotic and placebo groups, respectively. For Mazloom (2013) (3), the SD values should be 60.9 and 65.13 for the probiotic and placebo groups, respectively. Detailed calculation methods can be found in the statistical analysis section.
6. Supplementary Figure 4B (1): For Asemi(2013) (2), the SD values should be 2.03 and 1.51 in the probiotic and placebo groups, respectively. For Tonucci(2017) (4), the mean and SD values should be -0.67 and 1.46 in the probiotic group, and 0.31 and 1.17 in the placebo group. I also question why Wang et al. (1) did not include the study by Savytska (2023) (5) in the subgroup analysis for the 6-8 week intervention. Savytska’s study has an endpoint of 8 weeks, meeting the inclusion criteria, so I suggest adding this study to the subgroup analysis.
7. Supplementary Figures 4C–E (1): These figures appear to be identical, likely due to an oversight. In Supplementary Figure 4C (1), for Asemi(2013) (2), the SD values should be 5.15 and 6.91 for the probiotic and placebo groups, respectively. For Mazloom(2013) (3), the SD values should be 0.57 and 0.08 for the probiotic and placebo groups, respectively. In Tonucci(2017) (4), the mean and SD values should be -0.7 and 4.79 in the probiotic group, and -1.65 and 4.25 in the placebo group.
8. Supplementary Figure 4D (1): In Asemi(2013) (2), the mean and SD values should be 0.78 and 2.06 for the probiotic group, and 2.38 and 3.96 for the placebo group. For Mazloom(2013) (3), the mean and SD values should be -0.71 and 4.4 for the probiotic group, and 0.13 and 1.32 for the placebo group. For Tonucci(2017) (4), the mean and SD values should be 0.02 and 1.68 for the probiotic group, and 0.15 and 1.21 for the placebo group. Firouzi (2017) (6) reported mean and SD values of -0.4 and 1.8 for the probiotic group, and 0.9 and 2.0 for the placebo group.
9. Supplementary Figure 4E (1): In Kobyliak (2020) (7), the mean and SD values were -0.33 and 5.33 for the probiotic group, and 0.08 and 7.7 for the placebo group. For Razmpoosh (2019) (8), the mean and SD values were -0.3 and 4.2 for the probiotic group, and -0.1 and 4.2 for the placebo group. For Savytska (2023) (5), the mean and SD values were 0.03 and 0.48 for the probiotic group, and -0.08 and 0.59 for the placebo group. Firouzi (6) reported mean and SD values of -0.1 and 0.7 for the probiotic group, and 1.0 and 0.6 for the placebo group. Zikou (2023) (9) reported mean and SD values of -3.63 and 3.1 for the probiotic group, and -0.44 and 5.44 for the placebo group.

## Statistical analysis

All data were analyzed using RevMan version 5.3. For continuous variables, mean difference (MD) was used for those with uniform measurement units, while standardized mean difference (SMD) with a 95% confidence interval (CI) was used for those with differing units. The I² value was used to assess heterogeneity across studies, where values over 25%, 50%, and 75% represented low, medium, and high heterogeneity, respectively. When I² ≥ 50%, sensitivity or subgroup analyses were conducted, and the random-effects model was applied. When I² < 50%, the fixed-effects model was used. A p-value of <0.05 was considered statistically significant.

For the meta-analysis, changes in mean and SD from baseline to endpoint were analyzed between groups. If the final changes were not reported, the following methods were used: (1) If baseline and endpoint mean and SD values were provided, final changes were calculated using the formula SD = SQRT (SD1² + SD2² - (2 × R × SD1 × SD2), where R = 0.5 (10). (2) If the median and interquartile range (IQR) were reported, we approximated the mean ≈ median, and SD ≈ (P75 - P25)/1.35 (11, 12).(3) When standard error (SE) was provided, we calculated the SD using an online data calculator provided by the Cochrane website.here, SE refers to the standard error within the groups for both sets, so the SD for baseline and final can be calculated using the formula SD = SE ×√n (13). All results were rounded to two decimal places.

## Revised meta-analysis results

**Figure 1:**
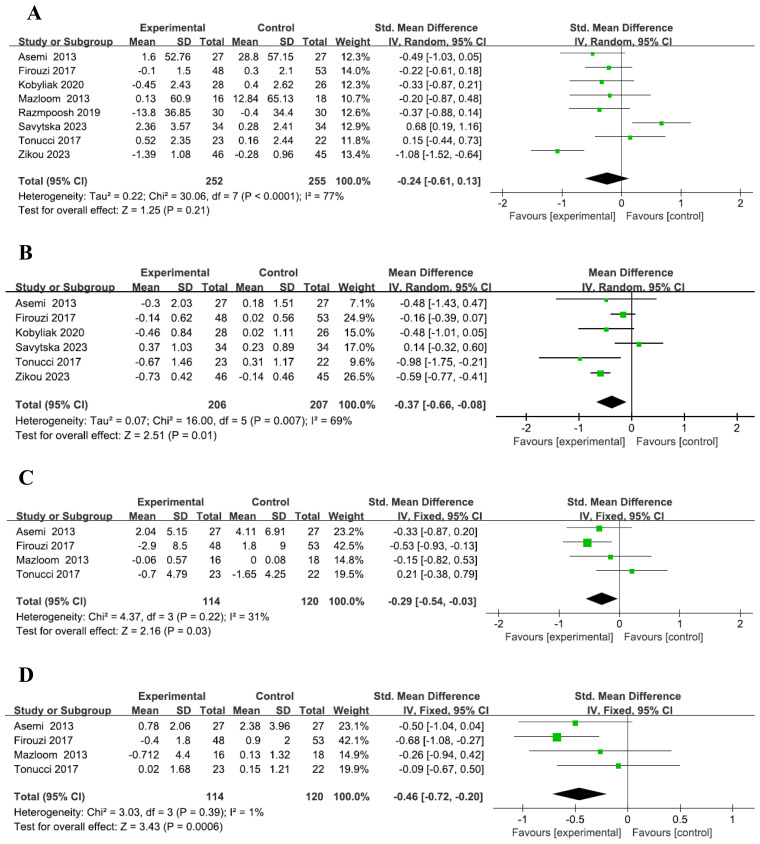
Forest plot of indicators. Forest plot of FBG level **(A)**. Forest plot of HbA1c level **(B)**. Forest plot of Insulin level **(C)**. Forest plot of HOMA-IR level **(D)**.

1. A total of eight studies were included (2–9), with 252 participants in the probiotic group and 255 in the placebo group. The reanalysis showed no significant difference in fasting blood glucose (FBG) changes between the probiotic and placebo groups, with high heterogeneity (SMD = -0.24, 95% CI: -0.61–0.13, P=0.21, I² =77%, **Figure 1A**). Subgroup analysis similarly found no significant difference between groups based on intervention time (**Supplementary Figure 1A**). These findings are consistent with those of Wang et al. (1).
2. Six studies (2, 4–7, 9), with 206 participants in the probiotic group and 207 in the placebo group, revealed that the probiotic group had a more significant reduction in HbA1c levels compared to the placebo group, with medium heterogeneity (MD = -0.37, 95% CI: -0.66– -0.08, P=0.01, I² =69%, **Figure 1B**). However, subgroup analysis showed no significant difference in HbA1c reduction across intervention times (**Supplementary Figure 1B**), which differs from Wang et al.’s findings (1).
3. A total of four studies were included (2–4, 6), with 114 participants in the probiotic group and 120 in the placebo group. The results showed a more pronounced reduction in insulin levels in the probiotic group compared to the placebo group, with low heterogeneity across the included studies (SMD = -0.29, 95% CI: -0.54 to -0.03, P=0.03, I² = 31%, **Figure 1C**). Subgroup analysis indicated that among patients with T2DM, a 12-24 week probiotic intervention led to a more significant decrease in insulin levels compared to the placebo group (SMD = -0.53, 95% CI: -0.93 to -0.13, P=0.09, **Supplementary Figure 1C**). These findings are consistent with those of Wang et al. (1).
4. Four studies were included (2–4, 6), with 114 participants in the probiotic group and 120 in the placebo group. The analysis revealed a more notable reduction in HOMA-IR in the probiotic group compared to the placebo group, with low heterogeneity among the included studies (SMD = -0.46, 95% CI: -0.72 to -0.20, P=0.0006, I² = 1%, **Figure 1D**). These findings are in line with those of Wang et al. (1), although the heterogeneity in our analysis was significantly lower. Subgroup analysis further demonstrated that in T2DM patients, HOMA-IR decreased more significantly in the probiotic group than in the placebo group after 12-24 weeks of probiotic intervention (SMD = -0.68, 95% CI: -1.08 to -0.27, P=0.001, **Supplementary Figure 1D**), which is consistent with Wang et al.’s findings (1).
5. Subgroup analysis, based on five studies (5–9), showed that after 12-24 weeks of probiotic intervention, T2DM patients experienced a significantly greater reduction in BMI in the probiotic group compared to the placebo group (SMD = -1.19, 95% CI: -2.14 to -0.25, P=0.01, I² = 89%, **Supplementary Figure 1E**). However, this contrasts with Wang’s study (1), which did not find a significant difference in BMI reduction between the probiotic and placebo groups over the same time period.

With the rapid pace of global industrialization and the sharp rise in obesity, Diabetes mellitus (DM) has become a prevalent metabolic disorder, primarily characterized by chronic hyperglycemia and accompanied by various complications (14). Among the types of diabetes, type 2 diabetes mellitus (T2DM) is predominantly caused by insufficient insulin production or secretion, coupled with chronic hyperglycemia due to insulin resistance. Typically diagnosed after the age of 40, T2DM constitutes approximately 90% of all diabetes cases, and the risk continues to increase with age (15). The risk factors associated with T2DM include hereditary factors, age, obesity, physical inactivity, gestational diabetes, poor diet, and stress (16). A meta-analysis conducted by Sun et al. demonstrated that probiotics could positively influence blood glucose regulation and offer benefits in both preventing and managing T2DM (17). Certain probiotic species have been found to enhance insulin sensitivity and decrease inflammatory markers (18). In our study, after adjusting the data, we observed that T2DM patients who received probiotic supplementation for 12 to 24 weeks showed a more significant reduction in blood insulin levels compared to those in the placebo group. This suggests that the improvement in insulin sensitivity among the probiotic group may explain these results. Other studies have shown that T2DM patients receiving stable metformin therapy, along with a probiotic formulation twice daily over a 12-week period, experienced substantial reductions in HbA1c and body weight compared to the placebo group (19). Further supporting evidence from a meta-analysis by Kaveh Naseri et al. indicated that probiotic supplementation in T2DM patients led to reductions in body weight and BMI, as well as improvements in lipid profiles (20). Similarly, Ding et al.’s meta-analysis revealed that probiotics significantly reduced tumor necrosis factor-α (TNF-α), C-reactive protein, and led to declines in fasting blood glucose (FPG), HbA1c, and HOMA-IR levels in T2DM patients (21).Our findings also corroborate these previous studies, as we observed more pronounced decreases in HbA1c, insulin, HOMA-IR, and BMI in the probiotic group compared to the placebo group by the end of the study.

In conclusion, probiotics may represent a promising adjunctive therapy for the treatment of T2DM.
