# Supplementary figures and images for: Commentary: Effect of probiotics at different intervention time on glycemic control in patients with type 2 diabetes mellitus: a systematic review and meta-analysis
Source: Front Endocrinol (Lausanne). 2025 Mar 19;16:1514969. doi: 10.3389/fendo.2025.1514969 (PMC11961410; doi:10.3389/fendo.2025.1514969)

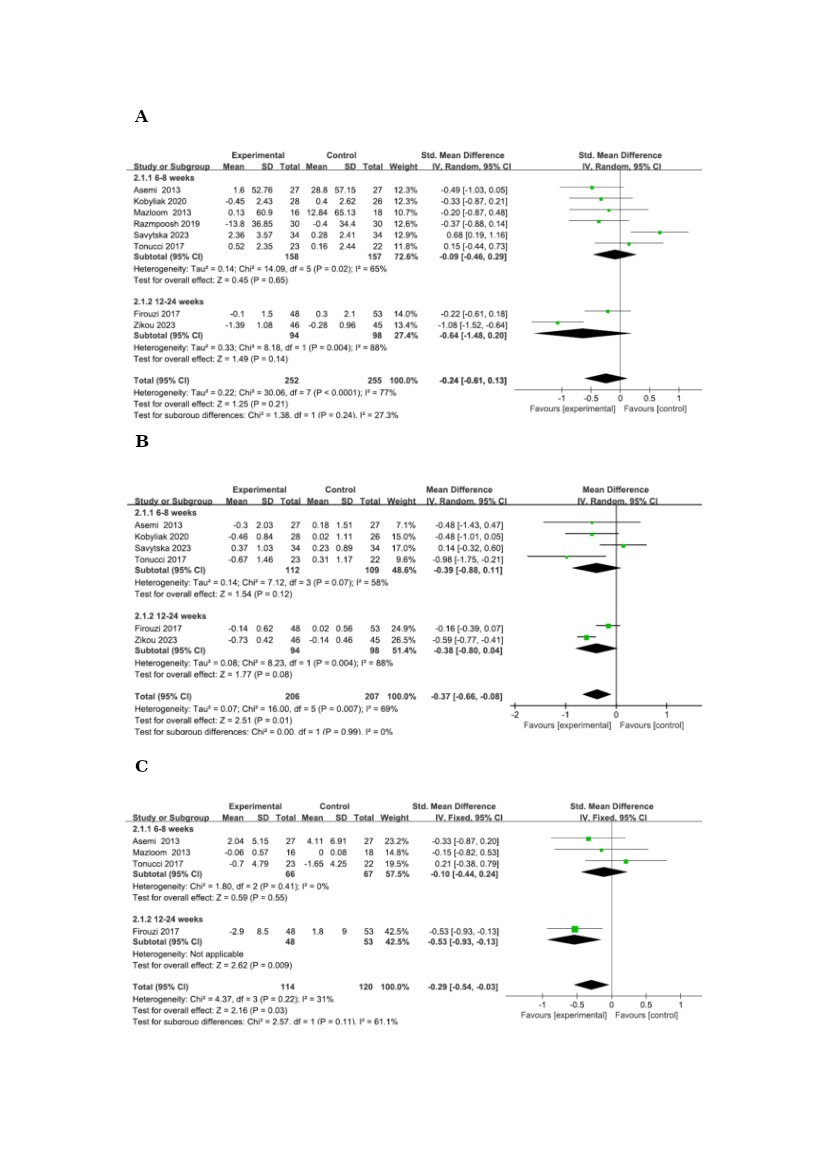

Supplement: Supplementary file 1 [file Image1.jpeg]

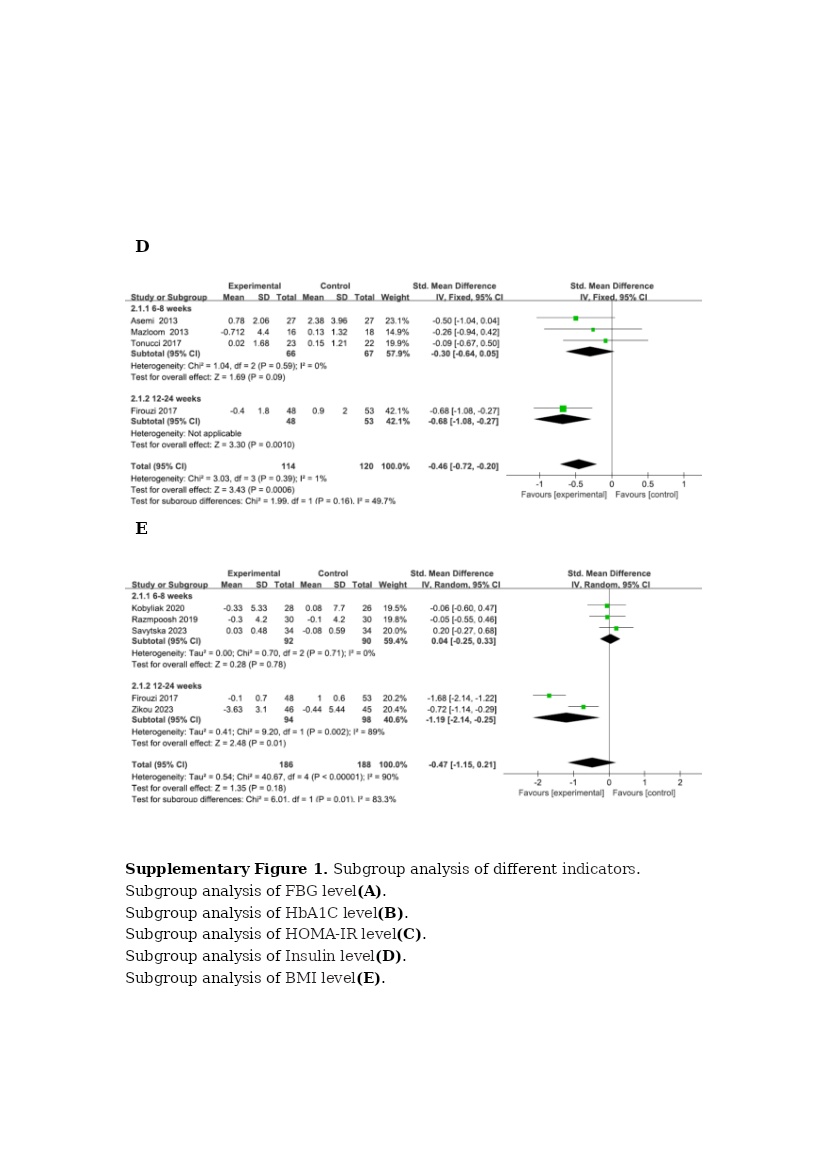

Supplement: Supplementary file 2 [file Image2.jpeg]
